# Collectanea

**Published:** 1828-05

**Authors:** 


### COLLECTANEA.

Floriferis ut apes in saltibus omnia libant,
Omnia nos, itidem, depascimur aurea dicta.

### ANATOMY.

*Observations on the Dissecting and Preparing of the Bodies of Animals.* By Professor Carus. (From his *Introduction to Comparative Anatomy*, translated by Gore, vol. ii. p. 389.)

Though the art of anatomising the bodies of animals is essentially the same as that practised upon the body of man, and though want of space precludes me from treating the subject minutely, I conceive that a few remarks may not be altogether unacceptable to those who feel desirous of pursuing such studies for themselves.

The first thing that I have to observe is, that all dissections of small and soft objects, *e.g.* worms, zoophytes, insects, mollusca, and embryos, where it is desirable to obtain even tolerably accurate results, should be performed *under water*, by which the parts are kept floating and separated from each other, and consequently present themselves more distinctly. A very simple contrivance for investigations of this kind may be prepared in the following manner:—A mass of tough wax (not too soft) is to be laid upon one or more porcelain saucers or capsules of different sizes, which are then to be put in a warm place until the wax melts so as to cover the surface evenly to the depth of a half or one-third of an inch. If the object to be examined be laid upon this surface, it may be fixed by needles in any position that is wished, and, when covered with clear water, developed and dissected by means of suitable instruments. Of them, the best are very delicate forceps; pointed, well made, sharp-cutting scissars; and small knives like cataract needles, some round, others with cutting edges, and fixed in slender wooden handles. For separating parts, I have also employed small horn probes and fine brushes; whilst, for examining them, a good magnifying glass is frequently indispensable. If it is wished to preserve a preparation thus made, wax, coloured at pleasure as for the purpose of injections, is to be formed into little tablets about one-fourth of an inch thick: one of these is then to be placed upon the saucer or capsule containing the preparation; the latter may then be transferred to it, arranged suitably upon it, fixed there by means of short needles, and both together then placed in alcohol. Nor must I forget to mention that the examination of very delicate organizations may frequently be conducted with greater facility and accuracy, if the object be previously allowed to remain some time in spirits, and thereby to become harder and contracted. This applies particularly to the dissection of nervous organs, and to the examination of very small embryos, of mollusca, and worms.

There are various modes of destroying worms, insects, mollusca, &c. for the purpose of dissecting, without injuring their organization. Mollusca, (snails, for instance,) as SWAMMERDAM has remarked, are to be allowed to die in water, because by that means their body swells, and all the parts become more distinctly visible; they may afterwards be kept in spirit (though not too long) for dissection. Worms, the larger zoophytes, (for the smaller must be examined whilst alive,) caterpillars, &c. and also the smaller amphibia and fishes, are best destroyed by means of spirit. Insects, on the contrary, by being dipped rapidly in boiling water, or in oil of turpentine.

As regards the dissection of larger animals, we may here use with advantage knives of a large size, and, instead of forceps, suitable hooks with handles.

In animals of considerable size we can generally make artificial skeletons only, after the bones have been sufficiently cleaned by boiling or maceration. In smaller animals, on the contrary, such as birds, amphibia, and fishes, of which last it is very difficult to make good skeletons, the object will be best accomplished by at once making the bones as clean as possible, without injuring the capsular ligaments, soaking the preparation in water that is incessantly changed, and, lastly, bleaching it for some time in the sun.

Lastly, we may mention injections as affording a very essential assistance in zootomical investigations for physiological purposes: in small animals, and in the more minute parts, these must consist of compositions with wax, very florid and coloured; but above all of mercury. The latter, however, is not suitable for very soft bodies, *e. g.* medusæ, &c. in which cases we may employ injections of coloured milk, and similar substances.

### PATHOLOGY.

Sir H. HALFORD *on Tic Douloureux.*—May I venture to throw out an opinion, founded on the observations with which my experience has furnished me, that the disease is connected with some preternatural growth of bone, or a deposition of bone in a part of the animal economy where it is not usually found in a sound and healthy condition of it, or with a diseased bone?

The following cases have occurred to me, and seem to give a degree of probability to this surmise; and I throw it out for the consideration of the profession, in order that a number of facts may be collected, from which a safe inference at length can be drawn.

A lady, forty years of age, suffered under the violent form of tic douloureux, at Brighton, notwithstanding the careful attention and skill of a very judicious physician there. On returning to town, it was observed that the rending spasms, by which the disease is marked, were frequently preceded by an uneasiness in one particular tooth, which exhibited, however, no signs of unsoundness; but the constancy of this symptom was enough to justify the extraction of the tooth in this instance, (though the failure of this expedient to afford relief in general does not encourage recourse to the operation,) and, on its being drawn, a large exostosis was observed at the root of the tooth, and the lady never suffered more than very slight attacks, and those very seldom, afterwards.

The D. of G. was attended by Dr. Baillie and myself, for six weeks, under this disease, in its most marked and painful form, without deriving benefit from our prescriptions. At length we thought it best to advise him to repair to the sea-coast, in hopes of renovating the shattered system by taking bark there. After he had sojourned a month by the sea-side, a portion of bone exfoliated from the antrum highmorianum, and the D. recovered immediately, and has never suffered the disease since. The bone had been hurt, probably, by a fall from his horse, which the D. had met with some months before.

The late Earl of C. underwent martyrdom by this disease, and excited the warmest sympathy of his friends by the agonies he sustained for many years. He submitted to the operation for the division of several branches of the fifth pair of nerves repeatedly, by Sir Everard Home and by Mr. Charles Bell, without obtaining more than mere temporary relief. At length he was seized by apoplexy, and lay insensible for some days, and in great peril from the attack, but finally recovered. After the apoplexy, the paroxysms of the tic douloureux became less frequent and less severe, and were administered so satisfactorily by an ingenious physician, who wrote his inaugural exercise on the disease. For the last year or two of his life, his lordship had ceased to suffer from the tic, and died at an advanced age, without any marked malady. His head was not examined after death, and therefore we are left to conjecture only what might have been the immediate cause of his former sufferings. Whilst I attended him, he underwent repeated exfoliations of the alveolar processes of the teeth, which I thought occasioned his torment; and, to account for the cessation of the complaint, I suppose that these efforts to throw off diseased portions of bone might have ceased, or that the apoplexy had disqualified the nerves for suffering so exquisitely; but there might have been besides, as some later instances have made probable, disease in the bones of the head.

The late Dr. P. fell a sacrifice to this dreadful disease, after sustaining its tortures for some years, with a constancy which attracted all our pity and esteem, and died at last under apoplexy. No assistance which the experience of any of us could afford him gave him relief, or controlled the violence of his attacks. On examining his head after death, there was found an unusual thickness of the os frontis, where it had been sawn through above the frontal sinuses, and at its juncture with the parietal bones. There was discovered also on the falciform process of the dura mater, at a little distance from the crista galli, a small osseous substance about three-eighths of an inch in length, rather less in breadth, and about a line in thickness. The vessels of the pia mater were turged with blood, and about an ounce of fluid occupied the ventricles. I lamented that the frontal sinuses had not been examined; for I remember he replied to a question which I once put to him, as to his ever having experienced any suppuration within any bony cavity, that he had twice suffered suppuration in the frontal sinuses. Dr. P. had submitted, with great patience, to a division of several branches of the fifth pair of nerves, under the judicious operation of Sir A. Cooper, who, on my mentioning to him the notion I entertained of the cause of tic douloureux, was so obliging as to show me the skull of a person who had died of this disease in the country. The internal surface of the frontal bone is a perfect rock-work. (*Medical Gazette.*)

---

***Death from swallowing two leaden bullets.***—Peritoneal inflammation resulted, and a fluctuation was felt. After death, one of the bullets was found near the anus in the pelvis, on the right side, at the termination of a fistulous canal, extending to the colon, which was perforated below. This canal contained pus and fæces. The bullet was evidently making its way into the rectum, which would have been accomplished had the patient survived.

An abscess also was formed and broke in the left groin, where the other bullet, in all probability, would have been found, had search been made for it.

This case is detailed by the editor of the *Philadelphia Journal.*

---

### PRACTICAL MEDICINE.

*Chorea cured by Iodine.*—Dr. Peltz relates an interesting case of this nature. He was led to use the Iodine from the following pathological views: He supposes the disease to be seated in part, if not altogether, in the tunica arachnoidea. It may be divided into acute and chronic. The acute consists in active inflammation of the above-named membrane, and the chronic in a thickening of the same, being a consequence of the acute stage, which yields to local and general active depletion, and the antiphlogistic regimen. It is to the chronic form that iodine is applicable.

In the case before us, Dr. P. directed fifteen leeches to each temple; salts, to procure free alvine evacuations; with pediluvium and sinapisms. The leeches and salts to be repeated every other day, and the pediluvium and sinapisms every day, for ten days. A blister was applied to the back of the neck, and kept open.

Frictions of turpentine were employed with some effect along the spine, and continued for some time. Under this treatment the improvement was slight. When Dr. P. thought that the chronic form had supervened, he prescribed Tr. Iodinæ in doses of six drops three times a day, which was gradually increased to twenty-four drops three times a day. Under this plan she improved, and at length completely recovered. (*Philadelphia Journal.*)

---

*Constipation.*—Dr. Parrish, of Pennsylvania, remarks that there is a form of constipation apt to be mistaken for diarrhœa. He says,

It occasionally happens that we are called to patients labouring under apparent diarrhœa, with frequent liquid evacuations, which in fact depend upon the collection of a mass of impacted and hardened fæces in the large intestines. The importance of forming a proper diagnosis must be obvious to every one; as treatment calculated to lessen or arrest the peristaltic action would certainly aggravate the disorder. In one of the cases recorded in the London Medical Observations, though, after long suffering, considerable relief was obtained by the discovery and removal of the offending cause, yet so much mischief had been previously produced, that the system of the patient sunk, and death ultimately resulted. The liability to mistake is increased by the insidious manner in which the complaint sometimes makes its approach. The patient has regular daily evacuations, a portion of the fæces being discharged, and a portion retained, till at length the accumulation becomes so great, and the mass so compact and hard, that the bowels are totally unable to overcome its resistance. But the passages, instead of being diminished, are now increased in number. The irritation of the mucous coat gives rise to augmented secretion; and the liquid, finding its way between the hardened mass and the side of the bowel, is discharged by stool. This happens more frequently as the irritation becomes greater, till at length the patient, believing himself to be affected with looseness of the bowels, and wearing away with the violence of the complaint, applies for medical advice. Should the physician, listening to his representations, mistake the nature of the affection and treat it as diarrhœa, the most serious consequences may be apprehended. An attentive examination of the symptoms will always enable him to form a correct judgment.

It will be readily conceived that, with such collections of offensive matter in the bowels, great abdominal uneasiness and irritation will be experienced; and frequent efforts will be made to procure relief by stool. These efforts, though generally productive of liquid discharges, are attended with much straining; and, as they are ineffectual in removing the offending cause, are not followed by entire relief. By this circumstance alone the complaint may be distinguished from diarrhœa, in which the pain always ceases for a time after the discharge. When the mass is situated in the colon, it sometimes happens that the symptoms of enteritis make their appearance; as tension of the abdomen, and great soreness upon pressure, attended with considerable febrile excitement. When the rectum is the seat of obstruction, the irritation occasioned by the pressure of the fæces is often extended to the bladder, and disease of this organ may be suspected as the sole cause of the symptoms. The same remark is applicable to the uterine system. In these cases, examination by the finger in the rectum will sometimes detect the real seat of disease; and the impossibility of throwing up injections, or their immediate return after they have been introduced into the bowel, affords additional evidence of the existence of obstruction.

The nature of the complaint having been ascertained, the treatment is exceedingly simple. Purgatives, assisted by injections, are the remedies chiefly to be relied on. I generally employ castor oil, or senna and salts, or jalap and cream of tartar. It is not sufficient to procure a few passages only: the cathartic should be repeated frequently, till hardened fæces cease to be discharged; and the quantity of dark-coloured offensive matter which thus comes away is sometimes so great as almost to exceed belief. When the feculent mass occupies the rectum, mechanical means, as the use of the finger or the handle of a spoon, may be brought in aid of the cathartic. The warm bath also will be occasionally found highly useful, by inducing relaxation; and general and local bleeding, with blisters to the abdomen, become necessary when symptoms of enteritis conjoin themselves with the constipation.

After the obstruction has been removed, its return should be obviated by the use of a laxative diet, assisted, if necessary, by an occasional resort to the milder cathartics.

One of the first cases which fell under my notice was that of a lady of this city. Some weeks after parturition, she was attacked with severe pain, and much general uneasiness in her abdomen, attended with frequent discharges from her bowels. When I first saw her, she told me that she was labouring under a "putrid lax;" a name she was induced to give to the complaint, in consequence of the extreme fetor of the evacuations. She was taking opium, port wine, and bark, with the view of strengthening her system, and checking the discharge. Finding, upon inquiry, that she had been affected in this way for a long time,—that her suffering was almost continual,—and that not even temporary relief was afforded by the evacuations,—I at once suspected the existence of hardened fæces in the bowels. The course of treatment she was before pursuing was immediately changed, and cathartic substituted for astringent medicine. By the free and repeated use of castor oil, which was happily well retained by the stomach, the bowels were thoroughly evacuated, and large quantities of feculent matter brought away, to the indescribable relief of the patient. She told me she suspected that the mass had remained in her intestines for five weeks; and that, when it was discharged, the first stools were so exceedingly offensive as almost to make her faint.

About the same time I was called to a little girl, in the family of a gentleman of Southwark, affected in a similar manner. She appeared to be labouring under diarrhœa; but no relief resulted from the evacuations: the pain and irritation were constant, considerable fever was present, and the symptoms of enteritis occurred during the course of the complaint. From these circumstances, suspecting the existence of feculent accumulations, I prescribed cathartics and injections, which were very freely administered. To overcome the inflammatory symptoms, several bleedings were necessary; a blister was also applied over the abdomen, and the warm bath employed. Her bowels at length yielded to the combined influence of these remedies, and large quantities of hardened fæces were discharged. After this she rapidly recovered.

I have known the complaint to occur in very young children. Not many years ago, I had under my care a little boy, the son of a highly respectable merchant of this city, who complained of great distress in his bowels, strained very much at stool, and, together with other liquid evacuation, passed a little blood. As the irritation was maintained notwithstanding the discharges, there was reason to suspect obstruction in the bowels; and the suspicion was confirmed by the fact that every effort to administer injections was rendered abortive by some impediment in the rectum. Thinking that the obstruction might be owing to hardened fæces, I passed my finger into the rectum, and found that such was really the case. By means of the finger, and the handle of a dessert spoon, I succeeded in breaking down the mass, and bringing most of it away. The patient was afterwards entirely relieved by purgatives. In this case I attributed the constipation to a diet almost exclusively of cow's milk. Another instance soon afterwards occurred to me, of a precisely similar character: the previous diet had been the same, and the symptoms yielded to the same treatment. In both instances, the mass which had occupied the rectum was found, when discharged, to consist of white indurated balls, bearing a close resemblance to soft white chalk. After the discharge of this matter, the patients were put upon a laxative diet, consisting of mush and molasses, with animal broths, to the entire exclusion of milk. In one of the cases, there was no return of the complaint; in the other, owing to the resumption of the former diet, there was a second attack, which, however, soon yielded to the usual measures. I have since seen several cases of constipation, with similar symptoms, originating in the same cause.

It occasionally happens that masses of indurated fæces are retained in the rectum, without producing any serious consequences. An old gentleman once applied to me on account of the constipated state of his bowels: he had experienced no great injury to his health; but his complaint was inconvenient, and he was naturally desirous of its removal. I prescribed cathartics, but there seemed to be some impediment which resisted the attempts at evacuation. I explained to him what I believed to be the nature of his case; and with his own finger he broke down the mass, and relieved himself of the inconvenience. Cases of a similar nature have since frequently occurred to me, generally among old people. (*North-American Med. and Surg. Journal.*)

### SURGERY.

*Case of Anchylosis.*—Dr. Barton has made some farther observations on the case of John Coyle, published in a recent Number of this Journal:

On the 19th of May, (says Dr. B.) he was discharged from the Pennsylvania Hospital, cured, using only a short cane. His freedom from hospital restraint tempted him to indulge in walking a great distance through the city, which caused him a return of pain and some inflammation. A few days of rest relieved him, and since that he has suffered no inconvenience. Some time in June, I made an unexpected visit to him in the vicinity of the city, and found that he had, just before my arrival, returned to the farm-house, with his cane in one hand, and a gun and some game in the other. I mention this incident to show that he not only had endured the fatigue of walking, but had sustained the jar accompanying the explosion of his piece with impunity.

I have recently made an examination of the hip, for the purpose of stating its present, and I suppose permanent, condition. There does not appear to have been any bony encroachment on the joint, but the ligamentous structure seems to have been so much thickened as to restrict, by its inelasticity, much of the motion of abduction and rotation. Adduction, flexion, and extension, appear to be unaffected; but, as any imperfection in the various movements is amply supplied by the latitude of motion between the lumbar vertebræ, it requires nice discrimination to detect, in walking, any want of free motion at the artificial joint; being able to project the foot in any direction. When he sits down, and the knees are placed in a line with each other, it will be found that the joint is flexed to its utmost, and that the curvature of the lumbar vertebræ contributes then to enable him to assume this position.

The patient declares that he never in exercise ascribes particular weakness to his hip, but that he endures fatigue there as well as in any other part of the body, and that he can walk ten miles without inconvenience.

Experience, founded on the treatment and result of this case, will, I think, induce me, on another occasion, to confine my patient for a longer time to his bed, in order to allow the newly formed ligaments to be perfected and strengthened. By this means it is probable that the erysipelatous attacks to which Coyle was subjected, may be avoided. The time also may be profitably employed in practising the movement of the joint. Advantage probably would be gained at this period of the case by changing the angle of the limb, and propping it in different positions for a day or two at a time. The eventual flexibility of the joint will, I think, be proportionate to the extent of movement during the curative process.

In contemplating this operation in such cases, the circumstance should not be overlooked in relation to its beneficial results, that it is not merely the loss of motion in a joint which constitutes the evil to be corrected, but it is also the mal-position of the limb. If, therefore, the operation should be performed, and the surgeon fail to effect the establishment of a new joint, in consequence of the tendency to ossific reunion, the lesion of the bone will enable him to place the limb in that position best suited for convenience and usefulness before consolidation shall again take place. The patient thus, in either event, will be compensated for his suffering, in the one instance by the services of a joint, or in the other by a removal of that distortion, which, in the lower extremity, would compel him to use crutches, or, in the upper, deprive him of many of the offices it would be capable of performing in a more favorable position of the limb.

An interesting and encouraging fact bearing upon this subject will be found in the perusal of Mr. SYME's case* of excision of the head of the humerus, in a carious condition. The glenoid cavity of the scapula was in a sound state, and the end of the humerus formed with it a joint susceptible of various movements by means of its own muscles.

The Periscopes of several of our own and of the foreign journals, contain a brief notice taken from a German periodical, (Gerson and Julius' Magazine for July and August, 1825,) of a joint formed also at the shoulder, by the removal of a spicula of bone projecting from the cavity of an ununited fracture. In this case it is said the muscles performed their respective offices. (*North American Med. and Surg. Journal.*)

---

*External Application of the Nitrate of Silver.*—Dr. BROWN, one of the physicians to the New-York Hospital, makes the following remarks upon the use of the nitrate of silver.

*Corns.*—Physicians are frequently asked the question, " what is good for corns?" and indeed it can hardly be otherwise, as almost all persons are more or less subject to them. Pressure is the sole cause of these troublesome excrescences, as it is of the ulcerated toe. They are generally produced by tight shoes and boots: hence many belles and beaus suffer greatly from them. But heavy persons are liable to corns, especially if they walk much, even though they wear loose shoes.

The formation of corns is doubtless a mere thickening of the cuticle, layer upon layer. They are painful and troublesome in proportion to their size. The pressure of the shoe or boot upon these hardened substances forces them in upon the cutis vera. This produces a constant irritation and an inflammation of the true skin, under and around the thickened cuticle; and, from the highly vascular and nervous structure of the skin, and the counter pressure of the bone and solid parts immediately beneath, produces that very acute pain which we so often suffer from corns. There is no difficulty in curing them ; neither should we have them, provided the cause, which is very evident, be removed. But, from feelings of pride in some, and from necessity in others, most persons suffer from them. It becomes then a subject of important information how to manage these troublesome excrescences while the cause continues to operate. We conceive the following plan the best: Bathe the foot at bedtime in warm water, until the corn becomes considerably softened. Shave the corn down with a knife or scalpel, but not so close as to draw the blood; then daub the part freely with the Nitrate of Silver, previously moistened with saliva or water, (the former is preferable.) The application should be extended a little beyond the borders of the corn, completely around, until such a quantity adheres as shall in a short time change it to a dark grey, and eventually completely black. There is no hazard of getting too much, especially upon the corn itself. Then wind a little raw cotton around the toe, and put on a stocking to confine it over the corn.

In about two or three weeks, the part acted upon by the caustic will slough off, including every vestige of corn, leaving the part quite smooth and of a natural appearance.

* See Edinb. Med. and Surg. Journal, vol. xxvi. 1826.

In this manner we have often removed corns which have not been succeeded by others in the same situation. They frequently, however, after awhile, grow again, from the operation of the ordinary cause, when they may be again removed in the same manner, and thus kept down with little trouble. Every individual, after having been once instructed, can manage his own case.

If the application be made at bedtime, which we advise, as it will afford several hours of complete relief from the operating cause, the tenderness and inflammation will be greatly abated, frequently completely relieved, by morning. The foot should be covered with cotton as long as any tenderness remains.

*Ulcerated Throat, Mouth, and Tongue.*—It is useless, perhaps, for us to speak of the good effects of the Nitrate of Silver in the venereal sore throat. It is doubtless the best local application, if used in a pure state. Dissolving in water renders it comparatively inert.

Several cases of ulcerated tongue have occurred under our observation, within a few years, of a peculiar nature. They were in persons of adult age, and mostly of a scrofulous or leuco-phlegmatic habit. The ulcers were scattered in different parts of this organ, and some far back at its base. They were of an aphthous appearance, some of which were quite deep, though not large, having inflamed edges. Several were of many months' standing, and had resisted various applications. We could trace them to no particular cause, but a peculiarity of constitution with a derangement of the digestive organs.

They were all healed in a very few days by the application of the lunar caustic.

The worst ulcerated tongue we ever beheld occurred to us a short time since, which was occasioned by an imprudent use of mercury in a peculiar habit of body. The tongue was ulcerated on each side, from one extremity to the other, and considerably thickened. The patient had not been able to eat, to thrust the tongue out of the mouth, or even to articulate, for three weeks. Her physician had directed the usual means, but amendment was so slow she thought proper to consult us. One free application of the caustic over the whole surface of the ulcers made a material alteration within twenty-four hours. It was applied daily, and within four days she could thrust the tongue from her mouth without any difficulty, as the swelling had greatly subsided; in eight or ten days she was completely cured.

The Nitrate of Silver appears serviceable in every grade of sore mouth.

I would remark upon the mode of applying the caustic within the mouth. As the parts are always moistened with the saliva, the stick should be perfectly dry. Touch the affected part, and remove the caustic instantaneously. If the part touched has received sufficient of its substance that it turns immediately white, it need not be applied again for that time:* if not, touch it a second time, and a third if necessary, till such effect be produced.

*Sore Nipples.*—Every practitioner who is much conversant with obstetric practice must have witnessed cases of this painful, and often obstinate and serious, affection. Serious, because it not unfrequently produces the necessity of taking the child from the mother's breast entirely, and sometimes occasions the obliteration of the orifice or destruction of the whole nipple, and of course renders it ever after useless. The ordinary means recommended in

* If the ulcer be small or deep, the stick should be brought to a point, or made somewhat conical, in order to carry it more surely to the bottom.

such cases often prove inefficient, or so slow in producing the desired effect as to induce mothers and nurses to abandon the prescriptions of the accoucheur, and to substitute their own or some hearsay remedy, which may be warmly recommended in such cases. Hence, under such a course of tampering, serious consequences are liable to occur, as above stated; and, indeed, they often prove extremely obstinate under the most judicious management.

The cause of this disease is very obvious, the stimulus of nursing the child in cases when the nipple has a peculiarly thin and tender cuticle. But we think such cases may generally be speedily cured, without removing the child from the breast; and that this may be effected by the application of the Nitrate of Silver. Having witnessed some troublesome affections of this nature, and been frequently disappointed in our prescriptions, and considering the efficacy of Nitrate of Silver in allaying erysipelatous inflammation, and in healing irritable ulcers of the cornea and other parts of the body, we tried it in the following case:

A lady, with her first child, had both nipples sore in three weeks after her confinement, which she attributed to the nipple glasses. Various applications were made with great care and attention; notwithstanding which they continued to grow worse for eight or ten days, when they became so exquisitely tender that the nursing of the child gave her excruciating torture. We proposed the application of the caustic, to which she assented.

At this time the top of the left nipple was in a state of ulceration, nearly surrounding the orifice. It appeared to be of the phagedenic character and extending. There were also several rhagades upon the sides of the nipple, running transversely, the longest of which was at the base. These fissures, whenever the child drew upon the breast, gave exquisite pain.

A stick of the Nitrate of Silver, with a conical point, was moistened and gently applied to the fissure, and to the edges of the ulcer upon the top of the nipple, so as to change the surface to a greyish colour.

The tenderness was less the following day, when it was applied twice, morning and evening. Two applications were also made the two succeeding days; after which, one only was made daily.

Within seven days the fissures were entirely healed, and the top nearly, so that nursing the child occasioned but little pain. The practice was pursued till they were completely healed, the child being continued at the breast the whole time.

The artificial nipple, made of the heifer's teat, expedites the cure if it can be used. In the above case, the child could not be made to suck it.

The question may naturally be suggested, whether it be perfectly safe for the child to suck the nipple to which the caustic has been applied? The affected part should be so lightly touched that but a small quantity adheres to the surface, which, combining with the substance of the skin, loses immediately its causticity, and becomes an inert substance, which, if taken into the child's stomach in a minute quantity, occasions no inconvenience.

We have also used the Nitrate of Silver with success in spongy gums, attended with a frequent oozing of blood, which were of long standing, and resisted other modes of treatment.

In chronic inflammation of the eyelids and cornea, it has proved effectual. It co-operates with nature in a truly wonderful manner, in restoring to healthy action parts which have been disorganised by wounds of different kinds, by ulcers and different grades of inflammatory action, particularly all character of unhealthy inflammation. If it be that kind of inflammation which attends aphthous affections of the mouth and throat, ulcers of the cornea, and various other cases of painful and unhealthy inflammatory action, it soon changes its character, and substitutes one which is less painful and of a healthy character, which disposes ulcers to heal.

In parts which want more action, as in lacerated and punctured wounds, and in ulcers of the phagedenic character and diminished action, it excites action to such a degree, and such only, as disposes them to healthy granulations.

It is equally favorable to ulcers and inflammation of the mucous surfaces, as in diseases of the skin; as in ulcers of the vagina and upon the glans penis, and in ulcers of the throat, mouth, and tongue. (*American Med. Recorder.*)

---

*Necrosis.*—The peculiar views of Professor Smith, and his mode of treating this disease, are contained in the following quotation:

"Necrosis, on the larger limbs, is somewhat analogous to the felon on the finger, where the parts beneath the strong fascia of the part are inflamed. In both cases a fibrous membrane is concerned, and, as in felon, an incision carried through the fibrous membrane to the extent of the inflammation stops the further progress of the disease; so, in necrosis, when the soft parts, with the periosteum, are divided, the disease is cured. The treatment, after the incision, both general and topical, should be such as we recommend in cases of simple incised wounds attended with considerable inflammation; excepting that we should not try to approximate the edges of the incision by adhesive plasters, but dress them with simple applications, such as lint spread with simple cerate, and evaporating lotions applied to a considerable portion of the limb, at least as far as the inflammation has extended. The general treatment consists in cooling purgatives, nauseating doses of antimony, and opium sufficient to allay irritation and procure rest."

He divides the disease into three stages: the first is inflammation of the bone and periosteum; the second when matter forms underneath the periosteum; and the third when the matter has escaped from beneath the periosteum, and is lodged in the surrounding soft parts. The first is to be treated by incision through the periosteum, the second by removing portions of the bone, by means of the trephine, after the incision has been made, so as to let out any matter which may form within the walls of the bone, thereby preventing its death. The last stage is mostly accompanied with a death of the bone, and requires the removal of the sequestra either by the efforts of nature or artificial interference. In describing the process, he gives some useful directions in relation to the operation, which we cannot detail. (*Philadelphia Journal.*)

---

*Cases, by* Dr. C. A. Weinhold, Professor at Halle. (From the *Bibliothek der Practischen Heilkunde.*)

I. *A large Callus of the left Femur, and a two-inch Shortening of the Limb, cured by Perforation and the Introduction of a Seton.*

John X. R., of eighteen years of age, broke, on the 21st of June, his left femur about its middle, and was treated in the usual manner by a neighbouring surgeon, till the expiration of the fourth week, when, at the earnest solicitation of his employer, he was permitted to resume his labours in the fields.

After a continued use of the limb for ten weeks, it became fully two inches shorter than the opposite one, and the callus acquired a size fully equal to that of a new-born child's head. At this time the sufferer was unable to work any longer, and therefore compelled to quit his employment, and to return to his destitute mother. There Dr. W. first saw and examined him, and found the callus to measure eighteen and a half inches in circumference. The cellular texture, both above and below the enlargement of the bone, was loaded with effused lymph, and in many places there were indurations of the soft parts, which ultimately suppurated and formed fistulæ. The disease was considered most unpromising, but, notwithstanding, the limb was placed in a proper apparatus, and extension by means of pulleys was made and persevered in for eight days, without the slightest effect upon the callus. Three months had now expired since the accident first took place, and the callus was so firm that no mechanical means whatever could effect its elongation.

In this state of things, the surgeon found himself obliged to abandon the patient to his miserable fate, or to resort to some new and untried expedient for his relief. He accordingly determined to perforate the callus, and pass through it a seton besmeared with stimulating substances, with the view of exciting inflammation and suppuration of the bone, and finally to bring about a softening and absorption of the callus, and, by a proper degree of regular extension, to restore the bone to its original length. To effect this, Dr. W., on the 11th November, in the presence of several medical gentlemen, adapted his needle-trephine to a turning bow, pierced the soft parts about an inch outside of the femoral artery, with the point of the instrument; which, on reaching the callus, was gently turned by the bow till it had perforated its external layers, and then it suddenly passed through a cavity about four inches deep before it reached the opposite side; which being bored in the same manner as the first, the point of the instrument was pushed through the muscle and integuments, and the seton introduced. The hemorrhage attending this operation was so trifling that it barely amounted to a single ounce.

During the first three days, cold poultices were applied to the parts. On the fourth and subsequent days, the seton, anointed with the Bals. Arceus, was moved through the wound twice each day. During the fifth week of the patient's confinement, the hardened cellular substance suppurated freely, the pus finding an exit by an opening above and below the callus. These fistulæ were cured by compression shortly after the induration of the cellular texture had disappeared.

About the sixth week there was great pain of the callus, and the temperature of the part was much increased : these symptoms yielded, in the course of forty-eight hours, to cold poultices.

In the seventh week the long-desired suppuration of the callus took place, and, on pressure with the finger, there was evidently a large chasm in it, indicating the propriety of the immediate application of the apparatus for extension : this, by the tenth week, produced such an elongation of the limb that it was barely two lines shorter than that of the opposite side.

For the sake of greater security, the seton was permitted to remain in the part till the twelfth week, at which time it was removed, and the orifice healed. A few weeks after, the callus was greatly diminished, the patient walked without crutches, and the diseased thigh was as large and almost as natural as the sound one. The man, after he had recovered his strength, was enabled to earn his livelihood as a coachman.

II. *Cure of artificial Articulation of the left Tibia, the ends of the bone being united by a fibrous structure, with a deep-seated suppuration of the calf of the leg.*

J. C. N., fifty-two years old, by profession a coachman, had the misfortune, in descending from the coach-box, to fracture his tibia, one and a half inches above its articulation with the foot. The next day the limb was properly attended to, and confined in a fracture-box; but, probably in consequence of too much motion, after a confinement of eight weeks, it was discovered that an artificial joint and a collection of matter in the calf of the leg had been formed. At the particular request of a medical friend, Dr. W. was induced to undertake the treatment of the case, according to his new mode of operating upon and of curing artificial joints. This method differs from that of Dr. Physick in its being more active and certain in its operation. Wardrop publishes cases in which Dr. P.'s plan failed entirely, and Hutchinson was compelled to withdraw his seton without effecting a cure.

After much reflection and observation, Dr. W. discovered that the inefficacy of the ordinary seton was owing to its exciting too slight an inflammation, and to its permitting the access of the external air to the long extremities; which, from that cause, are extremely disposed to become carious. Both these inconveniences are fully obviated by a funnel-shaped wound and a wedge-like seton.

In this case, a wound of an inch long was made upon the external part of the tibia down to the artificial joint, and a tape being previously passed through the eye of the needle-trephine, this was made to perforate the artificial joint, and pass on through the integuments of the opposite side, and then the tape, with the cuneiform seton attached, was drawn, till this last filled entirely the vacant space between the osseous extremities. The operation lasted at most but three minutes, and with the blood flowed a fetid pus, which became much more abundant upon pressure being made upon the calf of the leg. It was evident that the purulent collection communicated with the artificial joint, and of course the probability of the adhesive process taking place was materially lessened, and the hectic fever which already prevailed rendered the cure extremely doubtful.

After proceeding so far, it was altogether impossible to leave the work half done; therefore an incision was made into the abscess, from which a very large quantity of fetid ichor was discharged, and a colliquative suppuration followed, and nearly reduced the poor sufferer to the grave. Many physicians, at this unhappy time, recommended amputation as the only means of saving the patient's life; but, feeling the greatest confidence in the resources of the art, Dr. W. rejected the proposition, and succeeded, by proper bandaging and tonic medicines, not only in stopping the suppuration, but also, by the end of the thirteenth week, in curing the artificial joint without anchylosis or stiffness of the ankle-joint.

III. *Cure of an artificial Joint of ten years' standing, with Caries and Fistulæ, effected by a treatment of three months.*

J. C. H., a peasant, twenty years of age, when ten years old, fractured his right femur about its middle, and was treated in the usual manner by a country surgeon. At the end of the fourth week, whilst his parents were absent, he got up and used the limb; the consequence was, the formation of an artificial joint at the expiration of the eighth week. He, with the assistance of a stick, made out to move about with much difficulty, and was incapable of doing any duty but that of watching cattle, till he reached his nineteenth year, when, with the view of supporting himself, he engaged to do the duty of a stable boy, and soon, by his exertions, produced an inflammation of the artificial joint, which was succeeded by caries and a suppuration that formed two fistulæ posteriorly.

It was under these circumstances that he was taken to Dr. W. for his aid. Upon examination, the two fistulæ were found to terminate an inch from each other in the carious artificial joint. They were both thrown into one by an incision, and the cuneiform seton was introduced in the same manner and by the same means as in the foregoing case. A reaction speedily took place: the suppuration became more healthy, and each day carious portions of bone separated till the termination of the sixth week, when long splints were applied, and the part got firmer daily till the twelfth week, at which time, the bone bending no more, the seton was reduced in size each day, and finally withdrawn altogether. In three or four weeks after this, the patient could walk well, and was discharged perfectly cured.

IV.—*Fatal Attempt to cure an Artificial Joint by the Seton.*

A man, forty years of age, was thrown from a horse with his right hip against a stone, and fractured the neck of the femur, besides splintering the acetabulum. The surgeon, who was first called, considered the case as one of severe contusion, and ordered the part to be bathed with brandy. Three months after the accident, the patient was taken to Dr. W., who clearly ascertained that an artificial joint had been established, and, judging from the painfulness of the parts, he strongly suspected the existence of chronic inflammation and suppuration. The patient entreated the Doctor to attempt something for his relief, and the probability of success was deemed considerable, provided there should be no suppuration of the acetabulum or pelvis.

The sufferer was laid on a table, with the right hip projecting about four inches beyond its edge, and with the fingers the artificial joint was distinctly observable, about equidistant from the edge of the acetabulum and the great trochanter. An incision was made down to the artificial joint, and a large semicircular needle, armed with a tape and the cuneiform seton, was introduced from before backwards through it and the glutei muscles, till it passed out a little behind the great trochanter. The operation succeeded perfectly, and the patient complained of but little pain. Unfortunately, however, a putrid pus flowed from the wound, which, as it afterwards appeared, came from the carious acetabulum, and produced so active a hectic that the patient sunk, after suffering six weeks.

Upon inspection of the body, it appeared that the seton had passed directly through the artificial articulation, and that there existed a caries of the acetabulum, and a collection of matter in the pelvis.

V. *Introduction of a Seton in a Case of Spina Ventosa.*

A young man, of nineteen years of age, was afflicted with a spina ventosa of the middle of the left femur, with several fistulæ running backwards from the tumor, which measured about three inches in diameter. Amputation had been resolved upon as the only alternative; but the patient, being a waggoner, had determined to submit to any course at all likely to preserve his limb. Dr. W. proposed to him to unite the fistulæ, bore through the tumor, and pass into it the cuneiform seton: to this he very willingly assented, and the operation was performed in the same manner as those already described. After the expiration of three days, when the inflammation had subsided, the seton, previously anointed with a balsamic ointment, was introduced every morning and evening, and was so managed that it effectually closed the wound against the external air, and in the course of three weeks produced a good suppuration. The inflammation at first enlarged the tumor and produced considerable pain; but experience had taught Dr. W. that those were symptoms inseparable from the action of the seton, and that they were instrumental in exciting new life in the bone, which would resume, after the withdrawal of the seton, its healthy state. At the close of the thirteenth week the seton was removed, the apertures healed, and the bone speedily reduced to one-third of its former size.

---

*Successful Ligature of the Common Iliac Artery.* By VALENTINE MOTT, M.D. Professor of Surgery, New York.—ISRAEL CRANE, aged thirty-three years, by occupation a farmer, of temperate and regular habits, having generally enjoyed excellent health, says, about the middle of January, he felt some pain about the lower part of the belly, which he attributed to a fall received during the winter. He is in the habit of using great efforts in lifting heavy logs of wood, as his employment at this season consists in carrying wood to market. It, however, was not until a fortnight since, that he perceived any tumour about the lower part of the abdomen. Upon examination, the abdomen on the right side was considerably enlarged from about the crural arch, as high as the umbilicus. When the hand was applied to the parietes of the abdomen, a pulsation was felt and rendered visible to some distance. To the touch the tumour beat violently, and appeared to contain only fluid blood. It commenced a little above Poupart's ligament, and reaching, judging by the touch, from without, near the navel—inwards, almost to the linea alba—outwards and backwards filling up all the concavity of the ilium, and reaching beyond the posterior spinous process of that bone.

The rapid increase of this aneurismal tumour occasioned, as the countenance of our patient indicated, the most extreme agony. His sufferings at times were so great that his screams could be heard at a distance from the house. He had been bled several times, taken light food, and was kept constantly under the effect of opium. He was now informed of the serious nature of his case, and that without an operation very little chance of his life remained; with great composure he immediately consented to whatever would give him the best prospect of saving his life.

The pubes and groin of the right side being shaved, an incision was commenced just above the external abdominal ring, and carried in a semicircular direction half an inch above Poupart's ligament, until it terminated a little beyond the anterior spinous process of the ilium, making it in extent about five inches. The integuments and superficial fascia were now divided, which exposed the tendinous part of the external oblique muscle, upon cutting which in the whole course of the incision, the muscular fibres of the internal oblique were exposed; the fibres of which were cautiously raised by the forceps, and cut from the upper edge of Poupart's ligament. This exposed the spermatic chord, the cellular covering of which was now raised with the forceps, and divided to an extent sufficient to admit the forefinger of the left hand to pass upon the cord into the internal abdominal ring. The finger serving now as a director, enabled me to divide the internal oblique and transversalis muscles to the extent of the external incision, while it protected the peritoneum. In the division of the last-mentioned muscles outwardly, the circumflexa ilii artery was cut through, and it yielded for a few minutes a smart bleeding. This, with a smaller artery upon the surface of the internal oblique muscle, between the rings, and one in the integuments, were all that required ligatures.

With the tumour beating furiously underneath, I now attempted to raise the peritoneum from it, which we found difficult and dangerous, as it was adherent to it in every direction. By degrees we separated it with great caution from the aneurismal tumour, which had now bulged up very much into the incision. But we soon found that the external incision did not enable us to arrive to more than half the extent of the tumour upwards. It was therefore extended upwards and backwards about half an inch within the ilium, to the distance of three inches, making a wound in all about eight inches in length.

The separation of the peritoneum was now continued, until the fingers arrived at the upper part of the tumour, which was found to terminate at the going off of the internal iliac artery. The common iliac was next examined by passing the fingers upon the promontory of the sacrum, and to the touch appearing to be sound, we determined to place our ligature upon it about half way between the aneurism and the aorta, with a view to allow length of vessel enough on each side of it to be united by the adhesive process.

The great current of blood through the aorta made it necessary to allow as much of the primitive iliac to remain between it and the ligature as possible, and the probable disease of the artery higher than the aneurism, required that it should not be too low down. The depth of this wound, the size of the aneurism, and the pressure of the intestines downwards by the efforts to bear pain, made it almost impossible to see the vessel we wished to tie. By the aid of curved spatulas, such as I used in my operation upon the *innominata*, together with a thin, smooth piece of board, about three inches wide, prepared at the time, we succeeded in keeping up the peritoneal mass, and getting a distinct view of the arteria iliaca communis, on the side of the sacro vertebral promontory. This required great effort on our part, and could only be continued for a few seconds. The difficulty was greatly augmented by the elevation of the aneurismal tumour, and the interception it gave to the admission of light.

When we elevated the pelvis, the tumour obstructed our sight; when we depressed it, the crowding down of the intestines presented another difficulty. In this part of the operation I was greatly assisted by Dr. Osborn and my enterprising pupil, Adrian A. Kissam.

Introducing my right hand now behind the peritoneum, the artery was denuded with the nail of the forefinger, and the needle conveying the ligature was introduced from within outwards, guided by the forefinger of the left hand in order to avoid injuring the vein. The ligature was very readily passed underneath the artery, but considerable difficulty was experienced in hooking the eye of the needle, from the great depth of the wound and the impossibility of seeing it. The distance of the artery from the wound was the whole length of my aneurismal needle.

After drawing the ligature under the artery, we succeeded by the aid of our spatulas and board in getting a fair view of it, and were satisfied that it was fairly under the primitive iliac, a little below the bifurcation of the aorta. It was now tied—the knots were readily conveyed up to the artery by the forefingers—all pulsation in the tumour instantly ceased. The ligature upon the artery was very little below a point opposite the umbilicus.

The wound was now dressed with five interrupted sutures, passing them not only through the integuments, but the fibres of the cut muscles, so as to bring their divided edges together at all parts of the incision, which was muscular. Adhesive plaster to assist the stitches, lint and straps to retain it, completed the dressing. The operation lasted rather less than one hour.

He was removed from the table and put into bed upon his back, with the knee a little elevated upon pillows to relax the limb as much as possible, and to avoid pressure upon it. It was considerably cooler than the opposite leg, and flannels were applied all over it, and a bottle of warm water to the foot. From the habit he had been in of taking largely of anodynes, a tea-spoonful of the tinct. opii. was administered, with directions to repeat it in an hour if the pain should be severe.

In less than one hour from the operation, considerable reaction of the heart and arteries took place; he felt, as he stated, altogether relieved from the excruciating agony he had suffered since the aneurism commenced. The whole limb had now recovered its natural temperature.

*March 16th.*—The day after the operation, pulse eighty—skin moist—limb warm as the other—complains of some pain at the ligature—ordered a purgative of neutral salts.

17. Pulse eighty, and fuller than yesterday—took ℥x. of blood from his arm—skin moist—tongue brown—considerable uneasiness in the limb—no pain at the ligature—leg of natural heat—salts had a good effect.

*18th.* Pulse seventy-five—skin moist—tongue white—pain in the limb considerable—no pain at the ligature or in the wound—limb warm.

*19th.* Bled him today to ten ounces, the pulse being tense and beating eighty strokes in a minute—repeated the cathartic—suppuration appearing to have taken place, the dressings were removed.

*20th.* Pulse seventy and soft—skin moist—wound looks well—pain in the limb continues—leg warm as the other—cathartic operated well.

*21st.* Pulse seventy and soft—wound looks well—repeated the laxative—pain in the leg rather less—continues warm. There has been at no time tension of the abdomen or any particular uneasiness in that part. The patient thus far has been altogether more comfortable than could have been imagined. He takes more or less opium daily, from the long habit he has been in of taking anodynes.

*26th.* No unpleasant symptom—wound looks well—bled again to ℥xij. as there was a little tumefaction and inflammation about the wound.

*30th.* Our patient continues to do well—wound dressed daily.

*April 30th.* Is perfectly restored to health.

*May 29th.* My patient visited me today, having come twenty-five miles; he was so much improved in health that I did not recognise him. Examined the cicatrix and found it perfectly sound—could not discover any remains of aneurismal tumour—felt the epigastric artery much enlarged and beating strongly, and a feeble, though distinct, pulsation in the femoral artery immediately below the crural arch. The leg has its natural temperature and feeling, and he says it is as strong as the other.—*American Journal of Medical Sciences.*

*Note of a Case of Fistula in the Lumbar Region, communicating with the Bladder.* By L. Proudfoot, m.d.—E. D., aged twenty-four, applied to me on the 16th of March, 1827, on account of a fistulous opening which he had in the left lumbar region: upon examination with an elastic bougie, I found it entered about three inches straight forward, then taking a direction upwards, to the extent of eight inches, being within the parietes of the abdomen.

Mr. D. dates the commencement of the disease from a violent wrench, which he received at sea, while handing a topsail. This was immediately followed by a discharge of bloody urine and great prostration of strength, for several days.

I commenced the treatment by introducing a seton opposite the opening, and injecting a solution of Sulphas Cupri, of three grains to the ounce of water, internally; I prescribed the Murias Hyd. eight grains dissolved in eight ounces of brandy. Under this treatment his general health was much improved, and decidedly beneficial effects resulted from the injection, which was repeated every third day: the only uneasy sensation he felt was a slight pain in the left groin. About a fortnight after his application to me, he passed a considerable part of the injection from his bladder, which was followed by a discharge of wind from the urethra. This continued for several days, with a small discharge of urine from the opening in his back, particularly after getting up in the morning. He omitted taking the Murias Hyd., and I gave him the Bals. Copaiba in the dose of a tea-spoonful three times a day. Different injections were now employed; the black, yellow, and white wash were used, without any sensible difference in the depth of the opening.

The novelty of this case induced me to show it to my former preceptor and much esteemed friend, Dr. Mott, who strenuously recommended the use of the Oleum Terebinth.

About the beginning of October, I injected nearly two fluid drachms of the Oleum Terebinth. which soon after was voided by the bladder, with some discharge of flatus; since that time a great alteration has taken place, the depth of the fistula is at present about three inches, and the prospect of a recovery is now very flattering, the seton wore through the integuments in about three months, and the discharge from the opening has throughout the whole course of the disease, appeared perfectly healthy.

The injection was thrown to the top of the fistula by means of an elastic bottle, and a small catheter. (*Ibid.*)

### CHEMISTRY.

*Animal Matter in Mineral Waters.*—A green matter is deposited from the water of the hot alkaline springs of Vichy, in France. It was analysed by Vauquelin, who found it to resemble the white of an egg. It is worthy of remark, that springs in the south of France, and in the north of Italy, which issue from primitive rocks, should contain this substance, whose composition is so nearly the same as that of organic matter. (*Edinburgh New Philosophical Journal.*)

### MISCELLANEOUS.

*On the Irritability of the Sensitive Plant.*—M. Dutrochet has collected into a single volume the long and important researches which he has made upon the moving powers which act in organised bodies. His experiments on the sensitive plant occupy an essential part of this work. A new procedure, which he has employed in vegetable anatomy, has led him to results which would tend to invalidate a celebrated theory. He asserts that all the elementary organs of plants, that is to say, the cellules and tubes of which their body is composed, have an independent existence, and form circumscribed organs; so that these organs would only have to each other relations of vicinage, and would not form, by their assemblage, a really continuous tissue. He affirms that there are neither pores nor fissures visible to the microscope in the cellular tissue, any more than in the fibres of vegetables. There are only seen on the walls of these organs small semitransparent globular bodies, and linear bodies, which become opaque from the action of acids, and are rendered transparent by that of alkalies. M. Dutrochet considers these small bodies as the elements of a diffused nervous system. To the analogies of intimate structure and chemical nature, which he brings forward to support this opinion, the author adds physiological considerations, taken from experiments, which are peculiar to himself, and which, in his opinion, prove that the motions of vegetables are spontaneous: in other words, that they depend upon an internal principle, which immediately receives the influence of external agents. Refusing to admit *sensibility* in vegetables, M. Dutrochet substitutes for this term that of *nervimotility*.

With regard to the organ of motion in the leaves of the sensitive plant, M. Dutrochet has proved, by decisive experiments, that it consists in a bulging of the parenchyma, or of the *cortical medulla*, which is situated at the base of the petiole, and at the base of each of the leaflets of which the leaf is composed. He has discovered that this organ, to which he has given the name of *bourrelet*, is composed of globular cellules, disposed in longitudinal series, and filled with a coagulable fluid. It is not by means of joints that the sensitive plant, any more than the other irritable vegetables, moves its mobile parts; but by means of a curvature impressed on these parts in the place where the organ of motion occurs. Thus, in the sensitive plant, it is the bourrelets alone that, by curving, produce the folding of the leaves. M. Dutrochet has found that this curvature is the result of a vital elastic power, which even manifests itself in the thin slices that are taken from these bourrelets. He has given the name of *incurvation* to this phenomenon. Thus the vegetable irritability consists only in an elastic incurvation, which is sometimes fixed and sometimes oscillatory. For example, this elastic incurvation is fixed in the tendrils of vegetables, in the valves of the ovarium of the balsamine, &c.; it is oscillatory in the vegetables that are named *irritable*,—vegetables which present, in their mobile parts, a state of alternating incurvation and straightening.

It has long been known that the sensitive plant presents a phenomenon of sympathic transmission. If one of the leaflets of this plant be slightly burnt with a burning glass, all the leaflets belonging to the same stalk will fold themselves one after another. This motion deserves to be carefully examined; and, in order to determine the part of the stalk by which the transmission in question is operated, M. Dutrochet made several very delicate experiments, from which there results that it is neither produced by the pith nor the bark, but that it takes place exclusively by means of the woody part of the central system. Inquiring afterwards what, in this woody part, are the special organs of the transmission in question, he arrives at the conclusion of its being effected through the medium of the sap contained in the tubes which he names *corpusculiferous.* He has found that the maximum of velocity of this motion of transmission is fifteen millimetres per second in the petioles of the leaves, and only three millimetres per second in the body of the stalk. The state of the temperature does not appear to have any influence upon its velocity.

Light exercises a very remarkable influence upon the irritability of the sensitive plant, the observation of which equally belongs to M. Dutrochet. If a sensitive plant be placed in complete darkness, by covering it with an opaque vessel, it will entirely lose its irritability, and that in a variable time, according to a certain state of depression or elevation of the surrounding temperature. Thus, at a temperature of from twenty to twenty-five degrees of Reaumur, it requires only four days of darkness to destroy completely the irritability of a sensitive plant; while fifteen days of darkness are required to produce the same effect when the surrounding temperature is within the limits of ten and fifteen degrees; so that, on only taking the degrees of temperature in which the sensitive plant can live, it may be established that the extinction of the irritability of that plant in darkness is operated in a period, the duration of which is in the inverse ratio of the elevation of the temperature.

M. Dutrochet has observed that the sensitive plant, deprived of its irritability by means of darkness, recovers it by exposure to light; and that this restoring of the conditions of irritability is more rapidly effected by exposing the plant to the direct light of the sun, than by exposing it merely to the light of day, such as it exists in the shade. From these observations, M. Dutrochet considers light as the external agent from the influence of which vegetables draw the renewal of the conditions of their irritability, or, more generally, of their *motility;* conditions which are subject to deperdition in the natural state, and which thus require to be continually repaired. (*Edinburgh New Philosophical Journal.*)

---

*Recovery from Drowning.*—M. Bourgeois had occasion to give assistance in a case where, after a person had been twenty minutes under water, he was taken out, and, by a very common but serious mistake, carried with his head downwards. The usual means were tried unremittingly, but unsuccessfully, for a whole hour, but at the end of that time a little blood flowed from a vein that had been opened, and, a ligature being placed on the arm, ten ounces of blood were withdrawn: the circulation and respiration were then gradually re-established, horrible convulsions, and a frightful state of tetanus, coming on at the same time. Copious bleeding was again effected, after which a propensity to sleep came on; a third bleeding, the following morning, was followed by the recovery of the patient. Hence M. Bourgeois concludes, that the means of recovering a drowned person should never be abandoned until the decomposition of the body has commenced. (*Bull. Univ.*)

---

*An efficacious Means of promoting the Growth of Hair.*—A man, between twenty and thirty years of age, of strong and healthy constitution, having a short, curly, and coarse hair, of a dark brown colour, found himself becoming bald. Numerous and large bald spots appeared on the head, and gradually increased till it became perfectly naked, and, as the eyelashes fell out, the man had quite a singular and disagreeable appearance. When the head was closely examined, a short white and sperse down, very similar to a slight degree of mouldiness, was perceptible. At first it was hoped that the hair would grow again, but the sequel proved the contrary, for the individual remained in the same situation two years; when Dr. RADEMACHER advised him to pour French brandy upon the sulphate of copper, and, after it had remained a few days, to wash the bald parts with the solution once a day. In eight days the patient observed the hair had already begun to grow, and in four months it equalled in quantity the original growth, but it was crisp, dry, and stiff, and had not a natural appearance. On the back of the head a spot still remained bald, and the colour of the new hair was of a lighter cast than that of the former. The eyebrows and lashes grew again like the rest of the hair. A year after this the man shed his hair again, but the eyebrows and lashes remained. Dr. R. wished him now to await awhile, in order to ascertain whether it would or would not grow again spontaneously; but to this proposition the patient would not consent, and had recourse to the solution, which produced another growth of blond or light coloured hair; and the spot which before had continued bald notwithstanding the solution, became covered in common with the other parts of the head. This growth had a much more natural appearance than the preceding one. (*Bibliothek der Practischen Heilkunde.*)

